# Asymmetric autocatalytic reactions and their stationary distribution

**DOI:** 10.1098/rsos.231878

**Published:** 2024-10-23

**Authors:** Cameron Gallinger, Lea Popovic

**Affiliations:** ^1^ Department of Mathematics and Statistics, Concordia University, Montreal, Quebec H3G 1M8, Canada

**Keywords:** reaction networks, autocatalytic reactions, stationary distribution, discreteness-induced transitions, Moran model, genic selection

## Abstract

We consider a general class of autocatalytic reactions, which has been shown to display stochastic switching behaviour (discreteness-induced transitions (DITs)) in some parameter regimes. This behaviour was shown to occur either when the overall species count is low or when the rate of inflow and outflow of species is relatively much smaller than the rate of autocatalytic reactions. The long-term behaviour of this class was analysed in Bibbona *et al.* (Bibbona *et al.* 2020 *J. R. Soc. Interface* **17**, 20200243 (doi:10.1098/rsif.2020.0243)) with an analytic formula for the stationary distribution in the symmetric case. We focus on the case of asymmetric autocatalytic reactions and provide a formula for an approximate stationary distribution of the model. We show this distribution has different properties corresponding to the distinct behaviour of the process in the three parameter regimes; in the DIT regime, the formula provides the fraction of time spent at each of the stable points.

## 1. Introduction

Many important processes in biology rely on the switching behaviour between distinct states of the internal state of a system. Mathematically, this may be the result of alternating between different stable states for the dynamics of the molecular composition of the system. This feature is present in many gene-expression systems, where a gene alternates between two types of states (‘on’ and ‘off’) regulating the production of a protein. It is also present in many phosphorylation switches in signalling pathways. Such bistable switching patterns arise from distinct stable equilibria in the deterministic dynamics and the ability of infrequent large stochastic fluctuations to pull the system from a basin of attraction of one equilibrium to the other. However, there are also cases of chemical dynamics in which bistability is not possible in the deterministic model and is only possible in the stochastic model of the same chemical reaction system (e.g. [1]). The importance of stochastic effects in biological switching is well recognized, and a variety of stochastic processes are being used to model them (see [2] for a review).

Autocatalytic reaction networks are an important class of reaction networks whose long-term behaviour can substantively differ when modelled deterministically versus stochastically (e.g. [3]). Autocatalytic reactions are of broad interest in modelling living systems as they involve only a small number of distinct species and interactions, but they exhibit a range of outcomes such as self-sustaining growth, oscillations and symmetry breaking. Autocatalysis occurs in many elements of cellular metabolism including glycolysis, mitosis, apoptosis and DNA replication (for a review, see [4]) and may plausibly play a key role in the origin of life (e.g. [5]). They were recently used in chemical computing [6] as part of an artificial neural network design to perform digital image recognition tasks.

We analyse a model for a simple example of an autocatalytic reaction network proposed by Togashi & Kaneko [7]. They were particularly interested in the dynamics of the network when the total species count in the system was low and observed numerically that stochasticity at the low species count led to a new type of switching dynamics. The system spent most of its time in states where some combination of species is absent, switching rapidly by a successive increase in some species count from one such state to another. They called this behaviour discretely induced transitions (DITs) because discrete changes in species counts drive the switching. This dynamics is prominent in the parameter regime when the rates of autocatalytic reactions are fast relative to the rates of inflow and outflow from a reservoir, and the average time between switches is large.

The long-term behaviour of this model was further explored in [8], where the same authors observed that, if only the long-term averages are observed, autocatalytic reaction networks with strong symmetries do not show the important distinction between small and large species counts. However, in systems possessing asymmetry, numerical simulations clearly show that the shape of the long-term distributions shifts as the species count decreases. They observed similar effects in simple catalytic reaction networks in [9], where the low count of some species type effectively acts by switching on and off parts of the reaction network. They also observed this effect in spatial models in [10], where higher abundances are typically modelled by reaction–diffusion systems, but the localization and low abundance of some species types generate different possibilities for the long-term behaviour patterns of the system.

First, analytic results for symmetric autocatalytic reaction networks were obtained by a stochastic differential equation approximation of the system dynamics in [11]. They used a scaling parameter composed of the product of the system volume and inflow rate and derived an analytic expression for the stationary distribution of a linear combination of the species in the system, based on a timescale separation. In [12], they used the same tools to explore a version of this autocatalytic reaction network without inflows and outflows but with single species conversions. They provided a formula for the mean switching time between the states when the system contains only one of the species. A rigorous approximation by an obliquely reflected stochastic differential equation of a general autocatalytic reaction network was subsequently developed by Fan *et al*. [13], where a constrained Langevin approximation (based on the theory of Leite & Williams [14]) was analysed, together with the stationary distribution of this reflected stochastic differential equation. All of these results assume the system volume is large enough so that the limit as V→∞ is a useful approximation.

However, the validity of stochastic approximations is sensitive to differences in abundances in molecular amounts (e.g. [15]), as well as to separation of time scales in rates of reactions in the network (e.g. [16]). For this reason, it is more appropriate to analyse the properties of reaction networks from the Markov chain model directly. Analytic results for the Markov chain model were explored in [17], where the effect of species counts was shown on the flow of the molecular ‘current’ from one species type to another. Analytic results for the stationary distribution of a related class of models were derived in [18], for reaction networks without inflows and outflows and hence conserved overall species counts. A thorough analytic exploration of the autocatalytic reaction network with inflows and outflows was performed in [19], where it was shown that the Markov chain model of a general autocatalytic reaction network is exponentially ergodic. The authors further analysed the symmetric version of this model and derived an explicit form for the stationary distribution as a Dirichlet-multinomial distribution [19, theorem 4.3]. However, an explicit formula for the case of asymmetric autocatalytic reaction networks remained unknown.

To analyse the stationary distribution in the asymmetric case, we explored the connection of the autocatalytic model with inflows and outflows to that of the Moran model with mutation from population genetics. A connection with the Moran model was noted in [12,17] for symmetric rates of autocatalysis. We observe that asymmetric rates appear in Moran models that contain genic selection, as in [20], and the formula for the stationary distribution for such a Moran model is the starting point in our analysis. We explore the consequences due to the differences between the two models and explore how close the stationary distribution for the Moran model is to that of the autocatalytic network. We analyse the effect of the asymmetry in the long-term distribution of the species count in the system. We show the approximate stationary distribution also has the signature of the DITs in the parameter regime corresponding to low species counts or slow inflow and outflow rates in the system.

## 2. Stochastic (Markov chain) model for autocatalytic reactions

The autocatalytic reaction model we examine is the Togashi–Kaneko (TK) model for an open system. It consists of d molecular species $\{A_{i}{\}}_{i=1,\ldots ,d}$ involved in autocatalytic reactions and subject to inflows and outflows from the system:


(2.1) $$A_{i}+A_{j}\overset{\kappa_{i}}{\to }2A_{i}\;\;\emptyset \overset{\lambda_{i}}{\underset{\delta }{⇌}}A_{i}\;i,j=1,2,\ldots ,d.$$

The stochastic model is a continuous-time Markov chain $(X(t){)}_{t\geq 0}$, where $X(t)=(X_{1}(t),\;\ldots \;,X_{d}(t))$ counts the number of molecules of species i∈{1,…,d} present in the system at time t≥0. The state space for the Markov chain is $E=\{a=(a_{1},\ldots ,a_{d})\in {\mathbb{N}}^{d}\}$, and the transition rates are given by mass-action kinetics as


(2.2) $$\begin{array}{r} \begin{array}{rl} & q_{a,a-e_{j}+e_{i}}=\kappa_{i}a_{i}a_{j},\;\forall i\neq j\in \{1,\ldots ,d\}\\ & q_{a,a+e_{i}}=\lambda_{i}\;q_{a,a-e_{i}}=\delta a_{i},\;\forall i\in \{1,\ldots ,d\}. \end{array} \end{array}$$

Note that this is not necessarily a cyclic model (as all pairs of different species can catalyse each other), and the rates of autocatalytic reactions are different for each pair. The only constraint on the reactions that we impose is that the outflow rate is the same for all species, which ensures the lumpability of the Markov chain into a process that counts the total mass in the system.

The concept of *lumpability* means that the state space E can be partitioned into subsets $E_{n}=\{a\in {\mathbb{N}}^{d}|\sum_{i=1}^{d}a_{i}=n\}$, and that the *lumped* process $(N(t){)}_{t\geq 0}$ defined by $N(t)=\sum_{i=1}^{d}X_{i}(t)$ is a continuous time Markov chain on ℕ with associated transition rates


(2.3) $$\begin{array}{ccc} & q_{n,n+1}=\sum_{i=1}^{d}\lambda_{i}\;\;q_{n,n-1}=n\delta . & \end{array}$$

Because the total mass process $(N(t){)}_{t\geq 0}$ is a linear death process with immigration on ℕ, we know that it is irreducible and positive recurrent with stationary distribution ν that is Poisson with mean equal to $\mu :=\sum_{i=1}^{d}\lambda_{i}/\delta$ (e.g. example 4.5 in [21]).

In [19, theorem 4.1], Bibbona *et al.* showed that the Markov chain $(X(t){)}_{t\geq 0}$ describing the network (2.1) with transitions (2.2) is positive recurrent on E with a unique stationary distribution Π, and that it converges exponentially fast to this distribution.

In the special case when all the autocatalytic rates are the same, $\kappa_{i}=\kappa ,\forall i$, they derive an explicit formula for the stationary distribution Π in the form


(2.4) Π(a)=π(a|n)ν(n),

where ν∼ Poisson (μ) is the stationary distribution of the total mass process, and π(a|n) is the Dirichlet-multinomial (n,α) distribution on $E_{n}$:


(2.5) π(a|n)=(na)Γ(∑i=1dαi)Γ(n+∑i=1dαi)∏i=1dΓ(ai+αi)Γ(αi),

where (na)=n!/a1!⋯ad! for $a=(a_{1},\ldots ,a_{d})$, and parameters $\alpha =(\alpha_{1},\ldots ,\alpha_{d})$ are given by


(2.6) $$\alpha_{j}=\frac{\delta \lambda_{j}}{\kappa \sum_{j^{\prime }=1}^{d}\lambda_{j^{\prime }}}.$$

This analytic formula for Π from (2.4) and (2.5) is directly verified using the time evolution for the probability distribution of the system.

### 2.1. Connection to population genetics

The Moran model is a fundamental stochastic model for the evolution of a population of species types undergoing reproduction and mutation in continuous time (overlapping generations). The model describes a population of fixed size n, with d different species types $\{A_{i}{\}}_{i=1,\ldots ,d}$ , whose number of individuals at time t≥0 is denoted by $\tilde{X}(t)=({\tilde{X}}_{1}(t),\ldots ,{\tilde{X}}_{d}(t))$. Each individual of type i gives birth at the rate $\kappa_{i}$, and its offspring replaces an individual chosen at random to die. The species undergo mutations with each individual of type i changing to type j at a rate of $vp_{ij}$, where $p_{ij}$ are the entries of a stochastic matrix P and v>0 is the overall mutation rate.

The species reproduction and mutation changes can be represented graphically by


(2.7) $$A_{i}+A_{j}\overset{\kappa_{j}/n}{\to }2A_{j}\;A_{i}\overset{vp_{j}}{\to }A_{j},$$

where we assumed *parent-independent* mutations, $p_{ij}=p_{j}$. The fact that the fitness rates $\kappa_{j}$ are not equal across species is due to genic selection, and the special case $\kappa_{i}=\kappa ,\forall i\in \{1,\ldots ,d\}$ corresponds to a model that is neutral under selection.

The continuous time Markov chain of species counts $(\tilde{X}(t){)}_{t\geq 0}$ has transition rates from a to $a-e_{i}+e_{j}$ equal to


(2.8) $$\begin{array}{ccc} & {\tilde{q}}_{a,a-e_{i}+e_{j}}=a_{i}[\frac{\kappa_{j}}{n}a_{j}+vp_{j}]. & \end{array}$$

The Moran model differs from the model of autocatalytic reactions (2.1) in that it has a conservation of mass (closed system) and lacks the (open system) changes due to the inflow and outflow reactions. Comparisons of the long-term behaviour of closed versus open systems exist for deterministic models of reaction networks. If the deterministic open system is created from the deterministic closed system by adding inflow and outflow for each species type, then the open system has the same number of steady states and stability as the closed system [22–24]. For stochastic systems, the comparison is different as the closed system may have absorbing states when the open system does not.

The reason the long-term behaviour of the Moran model is remarkably close to that of the TK model is that one can create a correspondence between the mutation events in the Moran model and the inflows and outflows in the TK model (as explained in the next section). This correspondence does not couple the two models in a way that we can analytically exploit, but it has consequences that we can analyse numerically.

## 3. Approximate stationary distribution

To create a correspondence between the Moran model with genic selection and the autocatalytic reactions model, suppose we require that:

*each outflow of some species (this occurs at rate δ) is identified in time with the next inflow of some species (the probability that $A_{j}$ is the next species to inflow is$\lambda_{j}/\sum_{j^{\prime }=1}^{d}\lambda_{j^{\prime }}$*).

After such a modification, the autocatalytic reaction system becomes closed, and the rate at which each outflow event is simultaneous with an inflow event of species $A_{j}$ occurs equals $\delta \lambda_{j}/(\sum_{j^{\prime }=1}^{d}\lambda_{j^{\prime }})$. In the Moran model with genic selection, the rate of mutation of any species into species $A_{j}$ is $vp_{j}$. Hence, this forced correspondence of events transforms the autocatalytic reaction model (2.1) into a Moran model with genic selection (2.7), with the mutation rates given by the correspondence $vp_{j}=\delta \lambda_{j}/\sum_{j^{\prime }=1}^{d}\lambda_{j^{\prime }}$.

Although finite time effects of this forced correspondence cannot be rigorously analysed, we have the following long-time result for these Markov chains, in the special case when the rates of autocatalytic reactions in the TK model are symmetric and the Moran model is neutral.

**Lemma 3.1.** *Let* π(a|n) *be the stationary distribution of a TK model with symmetric autocatalytic rates* $\kappa_{j}=\kappa ,\forall j$ *conditioned on* $\sum_{i=1}^{d}a_{i}=n$*; let* $\pi_{n}(a)$ *be the stationary distribution of the neutral Moran model with* $\kappa_{j}=\kappa ,\forall j$*; and let the inflow and outflow rates* $\{\lambda_{j}{\}}_{j},\delta$ *in the TK model and the mutation rates* $\{p_{j}{\}}_{j}$ *in the Moran model satisfy* ∀j∈{1,…,d}*:*


(3.1) $$vp_{j}=\frac{\delta \lambda_{j}}{\sum_{j^{\prime }=1}^{d}\lambda_{j^{\prime }}}.$$

*Proof*. This fact is a direct consequence of identifying the two results on stationary distributions for the symmetric TK model and for the neutral Moran models, from [19] and [20] respectively. The Moran model with genic selection on a population of size n was analysed by Etheridge & Griffiths [20] and shown to have a stationary distribution proportional to


(3.2) πn(a)∝κ1a1⋯κdad(na)α1(a1)⋯αd(ad)|α|(n),a∈En,

where the parameters are given by


(3.3) $$\alpha_{j}=\frac{nvp_{j}}{\kappa_{j}},$$

$|\alpha |=\sum_{i=1}^{d}\alpha_{i}$, and $\alpha_{\left(a\right)}$ is the ascending factorial notation (Pochhammer function):


$$\alpha_{\left(a\right)}=\alpha (\alpha +1)\cdots (\alpha +a-1).$$

The normalizing constant for $\pi_{n}(a)$ is given as the partition function


(3.4) $$u\;(\alpha ,\kappa ,n)=E[(\sum_{i=1}^{d}\kappa_{i}\xi_{i}{)}^{n}],$$

where $\xi =(\xi_{1},\xi_{2},...,\xi_{d})$ has a Dirichlet-multinomial (n,α) distribution. Writing the ascending factorial in terms of Gamma functions $\alpha_{\left(a\right)}=\Gamma (\alpha +a)/\Gamma (\alpha )$, the stationary distribution can also be written as


(3.5) πn(a)∝∏i=1dκiai(na)Γ(∑i=1dαi)Γ(∑i=1dαi+n)∏i=1dΓ(αi+ai)Γ(αi).

In the case of the neutral model, when $\kappa_{i}=\kappa ,\forall i=1,\ldots ,d$, the partition function becomes $u(\alpha ,\kappa ,n)=\kappa^{n}𝔼\left[{\left(\sum_{i=1}^{d}\xi_{i}\right)}^{n}\right]=\kappa^{n}$ since $\sum_{i=1}^{d}\xi_{i}=1$. The stationary distribution then reduces to the Dirichlet-multinomial (n,α) distribution:


πn(a)=(na)Γ(∑i=1dαi)Γ(∑i=1dαi+n)∏i=1dΓ(αi+ai)Γ(αi),

which is the same as the stationary distribution π(a|n) from (2.5) derived by Bibbona *et al.* [19] in theorem 4.2 for which the Poisson–Dirichlet parameter α equals


(3.6) $$\alpha_{j}=\frac{\delta \lambda_{j}}{\kappa \sum_{j^{\prime }=1}^{d}\lambda_{j^{\prime }}}.$$

The requirement on mutation and inflow and outflow rates imposed in (3.1) completes the proof.∎

The long time effect of this correspondence in the symmetric case suggests the distribution $\pi_{n}$ from (3.5) may be a good candidate in forming the approximate conditional stationary distribution (conditioned on having $a\in E_{n}$) for asymmetric autocatalytic reaction system as well. We use ${\tilde{\pi }}_{n}$ to denote $\pi_{n}$ with parameters $\alpha_{i}$ satisfying (note the general rates $\kappa_{j}$ replacing κ from (3.6))


(3.7) $$\alpha_{j}=\frac{\delta \lambda_{j}}{\kappa_{j}\sum_{j^{\prime }=1}^{d}\lambda_{j^{\prime }}}.$$

We define our proposed approximate stationary distribution for the autocatalytic reaction system (2.1) as


(3.8) Π~(a):=ν(n)1u(α,κ,n)[∏i=1dκiai](na)Γ(∑i=1dαi)Γ(∑i=1dαi+n)∏i=1dΓ(αi+ai)Γ(αi),n=∑i=1dai.

We next analyse how well the distribution $\tilde{\Pi }$ approximates the true stationary distribution Π.

### 3.1. Analytic results

To assess how close $\tilde{\Pi }$ is to the true stationary distribution of (2.1), let A denote the generator of the Markov chain $(X(t){)}_{t\geq 0}$ defined for any f:E↦ℝ by


(3.9) $$\begin{array}{rl} Af(a) & =\sum_{i,j=1}^{d}q_{a,a-e_{j}+e_{i}}[f(a-e_{j}+e_{i})-f(a)]\\ & +\sum_{i=1}^{d}q_{a,a+e_{i}}[f(a+e_{i})-f(a)]+\sum_{i=1}^{d}q_{a,a-e_{i}}[f(a-e_{i})-f(a)], \end{array}$$

where the transition rates $q_{i,j}$ are as in (2.2). Let $p_{t}(m,a)$ denote the probability that the process $(X(t){)}_{t\geq 0}$ is in state a∈E at time t≥0, given that its initial distribution is X(0)∼m. Let $A^{*}$ be the adjoint of 𝒜 defined by


(3.10) $$\begin{array}{rl} A^{*}p_{t}(m,a) & =\sum_{i\neq j=1}^{d}[q_{a+e_{j}-e_{i},a}\;p_{t}(m,a+e_{j}-e_{i})1_{a+e_{j}-e_{i}\in E}-q_{a,a+e_{j}-e_{i}}p_{t}(m,a)]\\ & +\sum_{i=1}^{d}[q_{a+e_{i},a}p_{t}(m,a+e_{i})-q_{a,a+e_{i}}p_{t}(m,a)]+\sum_{i=1}^{d}[q_{a-e_{i},a}p_{t}(m,a-e_{i})-q_{a,a-e_{i}}p_{t}(m,a)] \end{array}$$

which specifies the evolution of $p_{t}(m,a)$ via the Kolmogorov forward equation (*chemical master equation* in the biology literature) as


(3.11) $$\frac{d}{dt}p_{t}\;(m,a)=A^{*}p_{t}\;(m,a)\;1_{a\in E}.$$

Since the Markov chain is irreducible and positive recurrent (see [19, theorem 4.1]), it has a unique stationary distribution **Π** on **$E={\mathbb{N}}^{d}$**. This stationary distribution Π of (2.1) is in the null space of $A^{*}$ and satisfies the *global balance equation*: $A^{*}\Pi (a)=0$. Using the proposed distribution $\tilde{\Pi }=\nu (n){\tilde{\pi }}_{n}(a)$, (3.10) becomes


(3.12) $$\begin{array}{rl} A^{*}\tilde{\Pi }(a) & =\nu (n)\sum_{i\neq j=1}^{d}[q_{a+e_{j}-e_{i},a}\;{\tilde{\pi }}_{n}(a+e_{j}-e_{i})-q_{a,a+e_{j}-e_{i}}{\tilde{\pi }}_{n}(a)]\\ & +\nu (n)\sum_{i=1}^{d}[q_{a+e_{i},a}\frac{\sum_{i}\lambda_{i}}{\delta (n+1)}{\tilde{\pi }}_{n+1}(a+e_{i})-q_{a,a+e_{i}}{\tilde{\pi }}_{n}(a)]\\ & +\nu (n)\sum_{i=1}^{d}[q_{a-e_{i},a}\frac{\delta \;n}{\left(\sum_{i}\lambda_{i}\right)}{\tilde{\pi }}_{n-1}(a-e_{i})-q_{a,a-e_{i}}{\tilde{\pi }}_{n}(a)], \end{array}$$

where ν∼ Poisson$\left(\sum_{i}\lambda_{i}/\delta \right)$ was used to express $\frac{\nu (n+1)}{\nu (n)}$ and $\frac{\nu (n)}{\nu (n-1)}$ in the second and third sums.

We now use $B^{*}(a):=A^{*}\tilde{\Pi }(a)/\tilde{\Pi }(a)$ to analyse how close $\tilde{\Pi }$ is to Π with **$B^{*}(a)\approx 0$** indicating good performance. Since for some values of system parameters (as discussed in the next section) **$\tilde{\Pi }$** concentrates on parts of the state space and has very low values on the rest of the state space, we chose to use the relative measure **$B^{*}(a)$** rather than **$A^{*}(a)$** as a potentially more reliable indicator of the closeness of **$\tilde{\Pi }$** to **Π**. By (3.12), we have


$$\begin{array}{l} \begin{array}{rll} B^{*}(a) & =\sum_{i\neq j=1}^{d}\left[q_{a+e_{j}-e_{i},a}\;\frac{{\tilde{\pi }}_{n}(a+e_{j}-e_{i})}{{\tilde{\pi }}_{n}(a)}-q_{a,a+e_{j}-e_{i}}\right]\\ & +\sum_{i=1}^{d}\left[q_{a+e_{i},a}\frac{\sum_{i}\lambda_{i}}{\delta (n+1)}\frac{{\tilde{\pi }}_{n+1}(a+e_{i})}{{\tilde{\pi }}_{n}(a)}-q_{a,a+e_{i}}\right] & +\sum_{i=1}^{d}\left[q_{a-e_{i},a}\frac{\delta \;n}{\sum_{i}\lambda_{i}}\frac{{\tilde{\pi }}_{n-1}(a-e_{i})}{{\tilde{\pi }}_{n}(a)}-q_{a,a-e_{i}}\right]. \end{array} \end{array}$$

For d=2, each sum has two terms which we gather (based on ± sign) into


$$\begin{array}{rl} & B^{*}(a)=\frac{1}{{\tilde{\pi }}_{n}(a)}[(L_{n-1}+L_{n}+L_{n+1})(a)-R_{n}(a)]\\ \text{with} & \\ & R_{n}(a)=[\lambda_{1}+\lambda_{2}+n\delta +(\kappa_{1}+\kappa_{2})a_{1}a_{2}]{\tilde{\pi }}_{n}(a),\\ & L_{n-1}(a)=\frac{n\delta \lambda_{1}}{\lambda_{1}+\lambda_{2}}{\tilde{\pi }}_{n-1}\;(a-e_{1})+\frac{n\delta \lambda_{2}}{\lambda_{1}+\lambda_{2}}{\tilde{\pi }}_{n-1}\;(a-e_{2}),\\ & L_{n}(a)=\kappa_{2}(a_{1}+1)\;(a_{2}-1){\tilde{\pi }}_{n}(a+e_{1}-e_{2})+\kappa_{1}(a_{1}-1)\;(a_{2}+1){\tilde{\pi }}_{n}(a-e_{1}+e_{2}),\\ & L_{n+1}(a)=\frac{\lambda_{1}+\lambda_{2}}{n+1}(a_{1}+1){\tilde{\pi }}_{n+1}(a+e_{1})+\frac{\lambda_{1}+\lambda_{2}}{n+1}(a_{2}+1){\tilde{\pi }}_{n+1}(a+e_{2}). \end{array}$$

We see here the effect of the inflow and outflow reactions that give rise to transitions from n to n±1, in different values of n in $\tilde{\pi }$ and the Poisson distribution ν. In the expression for $\tilde{\pi }$, the weighted Dirichlet-multinomial depends only on $a\in E_{n}$, but the normalizing factor u depends on n, so we use Gauss hypergeometric functions to simplify the ratios of $\tilde{\pi }$ appearing in $B^{*}$.

In terms of a Dirichlet-multinomial (n,α) variable $\xi =(\xi_{1},\ldots ,\xi_{d})$, the factor u is, by (3.4),


(3.13) $$u(\alpha ,\kappa ,n)=E[(\sum_{i=1}^{d}\kappa_{i}\xi_{i}{)}^{n}],$$

which we can also write, using the product moments of ξ, as


u(α,κ,n)=∑|a|=n[∏i=1dκiai](na)E[∏i=1dξiai]=∑|a|=n[∏i=1dκiai](na)Γ(∑i=1dαi)Γ(∑i=1dαi+n)∏i=1dΓ(αi+ai)Γ(αi).

For d=2, this can be further simplified using special functions. Using $a_{1}=i,a_{2}=n-i$,


u(α,κ,n)=∑i=0n(ni)κ1iκ2n−iΓ(α1+α2)Γ(α1+α2+n)Γ(α1+i)Γ(α1)Γ(α2+n−i)Γ(α2),

and the partition function becomes


u(α,κ,n)=Γ(α1+α2)Γ(n+α1+α2)Γ(α2+n)Γ(α2)κ2n∑i=0n(ni)(−1)i(α1)(i)(1−α2−n)(i)(κ1κ2)i=Γ(α1+α2)Γ(n+α1+α2)Γ(α2+n)Γ(α2)κ2n⁣2F1(−n,α1;1−α2−n;κ1κ2),

where ${,}_{2}F_{1}$ is the Gauss hypergeometric function, which when evaluated on a nonpositive integer in the first coordinate reduces to the polynomial  2F1(−n,x;y;z)=∑i=0n(−1)i(ni)(x)(i)(y)(i)zi.

The distribution ${\tilde{\pi }}_{n}$ on i∈{0,1,…,n} becomes


(3.14) π~n(i)=1u(α,κ,n)κ1iκ2n−i(ni)Γ(α1+α2)Γ(α1+α2+n)Γ(α1+i)Γ(α2+n−i)Γ(α1)Γ(α2)=1⁣2F1(−n,α1;1−α2−n;κ1κ2)(κ1κ2)i(ni)Γ(α1+i)Γ(α2+n−i)Γ(α1)Γ(α2+n).

Using (3.14) in the expressions $R_{n},L_{n},L_{n-1},L_{n+1}$ for $B^{*}$, we then get that (see appendix A)


(3.15) $$\begin{array}{ll} B^{*}(a)= & (\lambda_{1}+\lambda_{2})[1-\frac{{\;}_{2}F_{1}(-n,\alpha_{1},1-\alpha_{2}-n,\frac{\kappa_{1}}{\kappa_{2}})}{{\;}_{2}F_{1}(-1-n,\alpha_{1},-\alpha_{2}-n,\frac{\kappa_{1}}{\kappa_{2}})}]\\ & -\kappa_{2}(n-1+\alpha_{2})(\frac{a_{1}\alpha_{1}}{a_{1}-1+\alpha_{1}}+\frac{a_{2}\alpha_{2}}{a_{2}-1+\alpha_{2}})[\frac{{\;}_{2}F_{1}(-n,\alpha_{1},1-\alpha_{2}-n,\frac{\kappa_{1}}{\kappa_{2}})}{{\;}_{2}F_{1}(1-n,\alpha_{1},2-\alpha_{2}-n,\frac{\kappa_{1}}{\kappa_{2}})}-1]\\ & -\kappa_{2}(\frac{a_{1}\alpha_{1}}{a_{1}-1+\alpha_{1}}+\frac{a_{2}\alpha_{2}}{a_{2}-1+\alpha_{2}})[a_{1}(1-\frac{\kappa_{1}}{\kappa_{2}})-\alpha_{2}]-\kappa_{2}\frac{a_{1}\alpha_{1}}{a_{1}-1+\alpha_{1}}[\frac{\kappa_{1}}{\kappa_{2}}-1]\\ & -\frac{\lambda_{1}+\lambda_{2}}{n+\alpha_{2}}\frac{{\;}_{2}F_{1}(-n,\alpha_{1},1-\alpha_{2}-n,\frac{\kappa_{1}}{\kappa_{2}})}{{\;}_{2}F_{1}(-1-n,\alpha_{1},-\alpha_{2}-n,\frac{\kappa_{1}}{\kappa_{2}})}[a_{1}(\frac{\kappa_{1}}{\kappa_{2}}-1)+\alpha_{2}]. \end{array}$$

We use numerical properties of Gauss hypergeometric functions ${,}_{2}F_{1}$ to determine the assumptions on parameters $\lambda_{i},\kappa_{i},$ and δ that ensure $B^{*}(a)\approx 0$. We also investigate its dependence on the overall species count in the system. Note that in the symmetric case, $\kappa_{1}=\kappa_{2}$, simplifying $R_{n},L_{n-1},L_{n},L_{n+1}$ yields $B^{*}(a)=0$, ∀a, and hence $\tilde{\Pi }$ is the true stationary distribution for X (see proof of theorem 4.2 in [19]).

### 3.2. Scaling and numerical results

We let V denote the scaling parameter for the magnitude of the overall species count in the system (referred to as *volume* in previous analyses of the TK model) which in some scenarios one may consider to be large (i.e. V→∞). The classical mass-action scaling of the stochastic model for the autocatalytic reaction system (2.1) requires all bimolecular reaction rate parameters to scale as $V^{-1}$, all inflow rate parameters to scale as V, and all unimolecular outflow rate parameters to stay unscaled:


$$\kappa_{i}=\frac{\kappa_{i}^{\prime }}{V},\;\;\lambda_{i}=\lambda_{i}^{\prime }V,\;\;\delta =\delta^{\prime }\;\;\;\Rightarrow \;\alpha_{i}=V\alpha_{i}^{\prime }.$$

To simplify the expression for $B^{*}=A^{*}\tilde{\Pi }/\tilde{\Pi }$ and analyse its properties for low versus high molecular count V, we use the parametrization as in [19], with $D,\kappa_{i}^{\prime }∼O(1)$:


(3.16) $$\kappa_{i}=\frac{\kappa_{i}^{\prime }}{V},\;\;\lambda_{i}=DV,\;\;\delta =D,\;\;\;\Rightarrow \;\alpha_{i}=\frac{DV}{d\kappa_{i}^{\prime }}=V\alpha_{i}^{\prime }$$

and focus on the role of asymmetry in the autocatalytic rates $\kappa_{i}$ as well as on the effect of V. When d=2 we can rewrite $B^{*}$ from (3.15) in terms of scaled parameters (see appendix B) by factoring out the parameters DV and $\kappa_{1}^{\prime }/\kappa_{2}^{\prime }$ to get


(3.17) $$\begin{array}{l} \begin{array}{rl} B_{V}^{*}(a)= & dDV([1-\frac{F(-n,\frac{DV}{d\kappa_{1}^{\prime }},1-\frac{DV}{d\kappa_{2}^{\prime }}-n,\frac{\kappa_{1}^{\prime }}{\kappa_{2}^{\prime }})}{F(-1-n,\frac{DV}{d\kappa_{1}^{\prime }},-\frac{DV}{d\kappa_{2}^{\prime }}-n,\frac{\kappa_{1}^{\prime }}{\kappa_{2}^{\prime }})}]\\ + & \frac{1}{n+\frac{DV}{d\kappa_{2}^{\prime }}}\frac{F(-n,\frac{DV}{d\kappa_{1}^{\prime }},1-\frac{DV}{d\kappa_{2}^{\prime }}-n,\frac{\kappa_{1}^{\prime }}{\kappa_{2}^{\prime }})}{F(-1-n,\frac{DV}{d\kappa_{1}^{\prime }},-\frac{DV}{d\kappa_{2}^{\prime }}-n,\frac{\kappa_{1}^{\prime }}{\kappa_{2}^{\prime }})}[a_{1}(1-\frac{\kappa_{1}^{\prime }}{\kappa_{2}^{\prime }})-\frac{DV}{d\kappa_{2}^{\prime }}])\\ - & \kappa_{2}^{\prime }(n-1+\frac{DV}{d\kappa_{2}^{\prime }})[\frac{F(-n,\frac{DV}{d\kappa_{1}^{\prime }},1-\frac{DV}{d\kappa_{2}^{\prime }}-n,\frac{\kappa_{1}^{\prime }}{\kappa_{2}^{\prime }})}{F(1-n,\frac{DV}{d\kappa_{1}^{\prime }},2-\frac{DV}{d\kappa_{2}^{\prime }}-n,\frac{\kappa_{1}^{\prime }}{\kappa_{2}^{\prime }})}-1](\frac{a_{1}\frac{DV}{d\kappa_{1}^{\prime }}}{a_{1}-1+\frac{DV}{d\kappa_{1}^{\prime }}}+\frac{a_{2}\frac{DV}{d\kappa_{2}^{\prime }}}{a_{2}-1+\frac{DV}{d\kappa_{2}^{\prime }}})\\ - & \kappa_{2}^{\prime }[a_{1}(1-\frac{\kappa_{1}^{\prime }}{\kappa_{2}^{\prime }})-\frac{DV}{d}](\frac{a_{1}\frac{DV}{d\kappa_{1}^{\prime }}}{a_{1}-1+\frac{DV}{d\kappa_{1}^{\prime }}}+\frac{a_{2}\frac{DV}{d\kappa_{2}^{\prime }}}{a_{2}-1+\frac{DV}{d\kappa_{2}^{\prime }}})+\kappa_{2}^{\prime }\frac{a_{1}\frac{D}{d\kappa_{1}^{\prime }}}{a_{1}-1+\frac{DV}{d\kappa_{1}^{\prime }}}[1-\frac{\kappa_{1}^{\prime }}{\kappa_{2}^{\prime }}]. \end{array} \end{array}$$

From the analytical expression note that when $\lambda_{i}^{\prime }=\delta^{\prime }=D\ll \kappa_{i}^{\prime }$ then $B_{V}^{*}(a)=O(D)$ implies our proposed distribution $\tilde{\Pi }$ is close to the stationary distribution, regardless of whether the overall species count V is low or high. Intuitively, when inflow and outflow rates are small relative to the autocatalytic rates, the system spends most of its time with a constant overall species count, and hence the stationary distribution ${\tilde{\pi }}_{n}$ is accurate most of the time.

**Figure 1.**
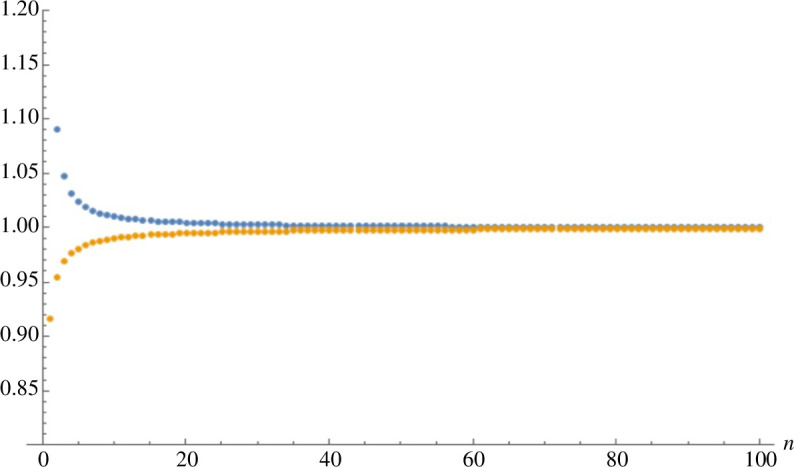
The ratios $\frac{{\;}_{2}F_{1}\left(-n,\alpha_{1},1-\alpha_{2}-n,\frac{\kappa_{1}}{\kappa_{2}}\right)}{{\;}_{2}F_{1}\left(1-n,\alpha_{1},2-\alpha_{2}-n,\frac{\kappa_{1}}{\kappa_{2}}\right)}$ and $\frac{{\;}_{2}F_{1}\left(-n,\alpha_{1},1-\alpha_{2}-n,\frac{\kappa_{1}}{\kappa_{2}}\right)}{{\;}_{2}F_{1}\left(-1-n,\alpha_{1},-\alpha_{2}-n,\frac{\kappa_{1}}{\kappa_{2}}\right)}$ plotted in blue and orange, respectively, as a function of *n*. The rate parameters are $\lambda_{1}=\lambda_{2}=2,\;\delta =0.01,\;\kappa_{1}=1,\;\kappa_{2}=1.001$.

**Figure 2.**
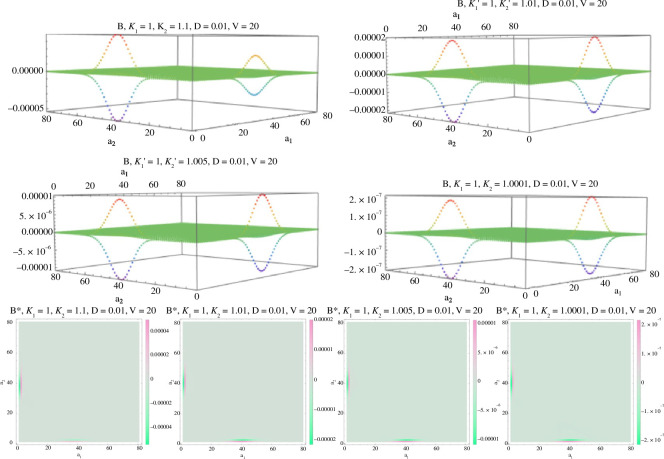
$B^{*}$ plotted with $\lambda_{1}^{\prime }=\lambda_{2}^{\prime }=\delta^{\prime }=0.01,V=20$, $\kappa_{1}^{\prime }=1$ and $\kappa_{2}^{\prime }=1.1,1.01,1.005,1.0001$ gradually decreasing $\kappa_{1}^{\prime }/\kappa_{2}^{\prime }\to 1$ from top left to bottom right. The value of $B^{*}(a)=0$ for all a except at the values $a_{1}=0$ or $a_{2}=0$ where the occupation measure concentrates (see figure 3). The bottom row are heatmaps of the exact same figures from the top two rows, with **$\kappa_{1}^{\prime }/\kappa_{2}^{\prime }\to 1$** from left to right; the only **$B^{*}(a)\neq 0$** values are at points **a=(0,dV),(1,dV)**, **a=(dV,0),(dV,1)**. Light neutral green in the square denotes **0**; the only deviations are on the left and bottom edges.

Numerical explorations show the two ratios of the Gauss hypergeometric functions appearing in $B_{V}^{*}$ are both close to 1 for all $\left(a_{1},a_{2}\right)$ (figure 1), so the two terms in the large square brackets are small. Further, numerical evaluations of $B_{V}^{*}(a)$ for different values of $\left(a_{1},a_{2}\right)$ are all close to zero (figure 2). The very small deviations from zero occur only when: $\left(a_{1},a_{2})\in \{(0,dV),(1,dV),(dV,0),(dV,1)\right\}$. These are values on the boundaries of the state space E with the total amount of species $n=a_{1}+a_{2}\approx dV$ close to the mean of the stationary distribution of the total species count (ν∼Poisson ($\sum_{i}\lambda_{i}^{\prime }/\delta^{\prime }=dV)$). At the two boundaries, $a_{1}=0$ and $a_{2}=0$, only outflows and inflows occur, and potentially the need to adjust the accuracy of the stationary distribution ${\tilde{\pi }}_{n}$ there. Changing $\kappa_{1}^{\prime }/\kappa_{2}^{\prime }$ while keeping all other parameters fixed, the deviations from zero disappear as $\kappa_{1}^{\prime }/\kappa_{2}^{\prime }\to 1$ (see figure 2) as expected.

**Figure 3.**
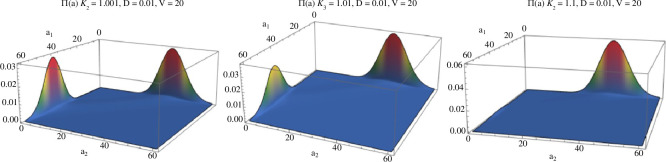
Plots of the analytic formula for $\tilde{\Pi }(a)$ when DV=0.2<d=2 show bimodal density. Parameters $D=0.01,V=20,\kappa_{1}^{\prime }=1$ are fixed while $\kappa_{2}^{\prime }$ is increased in plots from left to right: $\kappa_{2}^{\prime }=1.001,1.01,1.1$. Note the modes occur at $\left(a_{1},a_{2})\in \{(0,dV),(dV,0)\right\}$ with dV=40.

We further examine the features of ${\tilde{\Pi }}_{V}$ by noting the effect of scaling on ${\tilde{\Pi }}_{V}$ is reflected both in $\nu_{V}∼$Poisson ($\sum_{i}\lambda_{i}^{\prime }/\delta^{\prime }=dV$) and in the scaling of Gamma functions in ${\tilde{\pi }}_{n,V}$ (the V scaling of $\kappa_{i}^{\prime }$ in pre-factor cancels with same in $u_{V}$):


π~n,V(a)=1uV(α′,κ′,n)(na)[∏i=1d(κi′)ai]Γ(V∑i=1dαi′)Γ(n+V∑i=1dαi′)∏i=1dΓ(ai+Vαi′)Γ(Vαi′),uV(α′,κ′,n)=Γ(∑i=1dVαi′)Γ(n+V∑i=1dαi′)∑|a|=n(na)∏i=1d(κi′)aiΓ(Vαi′+ai)Γ(Vαi′).

For d=2, we can also write the scaled ${\tilde{\pi }}_{n,V}$ using a=(i,n−i) in terms of Beta functions:


(3.18) π~n,V(i)=1uV(α′,κ′,n)(κ1′)i(κ2′)(n−i)(ni)B(i+DVdκ1′,n−i+DVdκ2′)B(DVdκ1′,DVdκ2′),

as a weighted Beta-binomial distribution. Just as in the symmetric case (see [19, §4.3]), our proposed distribution ${\tilde{\Pi }}_{V}=\nu_{V}{\tilde{\pi }}_{n,V}$ will have a different shape: unimodal or multimodal, depending on whether: DV<d or DV>d.

When DV<d the parameters $\alpha_{1}^{\prime }=DV/(d\kappa_{1}^{\prime }),\alpha_{2}^{\prime }=DV/(d\kappa_{2}^{\prime })$ in the Beta-binomial are both <1, the weights have a small effect, and the distribution is bimodal. Since $\alpha_{2}^{\prime }/\alpha_{1}^{\prime }=\kappa_{1}^{\prime }/\kappa_{2}^{\prime }<1$, the mode at the value $\left(a_{1},a_{2})=(0,n\right)$ is higher than at $\left(a_{1},a_{2})=(n,0\right)$. Note that even very small asymmetries, e.g. $\kappa_{2}^{\prime }=1.0001,\;\kappa_{1}^{\prime }=1$, show a sizeable asymmetry in the size of the modes, and for a greater ratio, e.g. $\kappa_{2}^{\prime }=1.1,\kappa_{1}^{\prime }=1$, almost the entire mass of the distribution is concentrated at the higher mode (see figure 3). Of course, on each plane (0,n) and (n,0) there is a mode as well at dV from the distribution of total molecular counts $\nu_{V}$.

**Figure 4.**
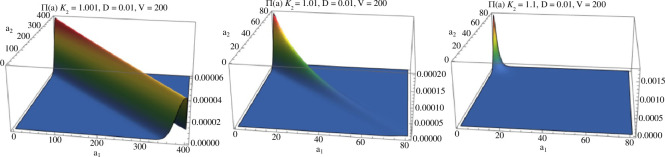
Plots of $\tilde{\Pi }(a)$ when DV=d=2. Parameters are $D=0.01,V=200,\kappa_{1}^{\prime }=1$, and $\kappa_{2}^{\prime }$ increases in plots from left to right: $\kappa_{2}^{\prime }=1.001,1.01,1.1$.

In the intermediate case, when DV=d=2 we have $\alpha_{1}^{\prime }=1$ and $\alpha_{2}^{\prime }=1/\kappa_{2}^{\prime }$. If $\kappa_{2}^{\prime }=1$ the evaluation of $\tilde{\Pi }$ on each hyperplane $E_{n}$ would put equal mass on {(0,n),…,(n,0)}, but for $\kappa_{2}^{\prime }>1$, it becomes skewed towards {(0,n)}, when e.g. $\kappa_{2}=1.1$ almost all the mass is at (0,dV=400) (see figure 4).

When DV>2, we have $\alpha_{1}^{\prime },\alpha_{2}^{\prime }>1$ and $\tilde{\Pi }$ is unimodal concentrated around the fixed point $\left(a_{1}^{*},a_{2}^{*}\right)$ of the deterministic system for this model


$$\begin{array}{r} \begin{array}{rl} \frac{d}{dt}a_{1}(t) & =(\kappa_{1}^{\prime }-\kappa_{2}^{\prime })a_{1}a_{2}+\lambda_{1}^{\prime }-\delta^{\prime }a_{1}\\ \frac{d}{dt}a_{2}(t) & =(\kappa_{2}^{\prime }-\kappa_{1}^{\prime })a_{1}a_{2}+\lambda_{2}^{\prime }-\delta^{\prime }a_{2}. \end{array} \end{array}$$

With $\kappa_{1}^{\prime }=1$, increasing $\kappa_{2}^{\prime }>1$, the mode moves such that the distribution is concentrated at a higher proportion of $a_{2}$ species to $a_{1}$ species. When $\kappa_{1}^{\prime }=\kappa_{2}^{\prime }$, this is a simple linear first-order ODE with fixed point$(a_{1}^{*},a_{2}^{*})=(\lambda_{1}^{\prime }/\delta^{\prime },\lambda_{2}^{\prime }/\delta^{\prime })=(V,V).$

**Figure 5.**
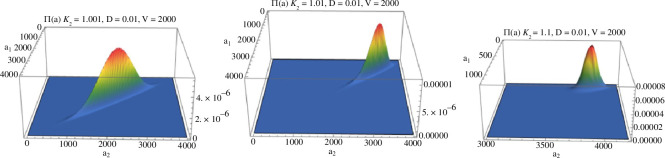
Plots of $\tilde{\Pi }(a)$ when DV=20>d=2. Parameters are $D=0.01,V=2000,\kappa_{1}^{\prime }=1$, and $\kappa_{2}^{\prime }$ increases in plots from left to right: $\kappa_{2}^{\prime }=1.001,1.01,1.1$.

However, when $\kappa_{1}\neq \kappa_{2}$, this system of ODEs is nonlinear, and we found numerically for $\kappa_{2}^{\prime }=1.001,1.01,1.1$ that the fixed points are, respectively, $\left(a_{1}^{*},a_{2}^{*})=(1801.96,2198.04),(763.93,3236.07),(97.5,3902.5\right)$ (figure 5, left to right).

**Figure 6.**
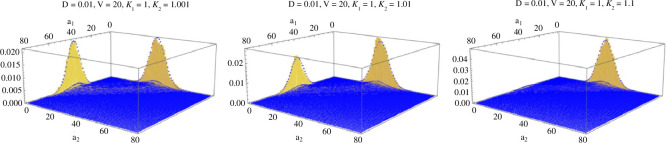
Histograms for the fraction of occupation times in all states a of X are shown in yellow with $\tilde{\Pi }(a)$ overlaid in blue. The parameters are $\lambda_{1}^{\prime }=\lambda_{2}^{\prime }=\delta^{\prime }=D=0.01,V=20,\kappa_{1}^{\prime }=1$, varying $\kappa_{2}^{\prime }=1.001,\;1.01,\;1.1$ from left to right.

Since the Markov chain $(X_{t}{)}_{t\geq 0}$ is ergodic, the long-term fraction of its occupation times converges to its stationary distribution. We simulated the Markov chain, using the Gillespie algorithm, to further compare our approximate stationary distribution $\tilde{\Pi }$ with the occupation time when DV<d. In that case, the modes at the boundaries of E reflect the DIT nature of the system, where most of the time is spent in the states $\{(0,n),(n,0){\}}_{n\geq 0}$, and the quick transitions between these states results in the lack of occupation time spent in the interior of E. As the asymmetry $\kappa_{2}^{\prime }/\kappa_{1}^{\prime }>1$ is increased, the DITs become more and more rare, as the autocatalytic reaction $A_{1}+A_{2}\overset{\kappa_{2}}{\to }2A_{2}$ overpowers the autocatalytic reaction in the opposite direction. Figure 6 shows the empirical long-term occupation time of the simulated Markov chain X along with analytic plots of $\tilde{\Pi }$.

**Figure 7.**
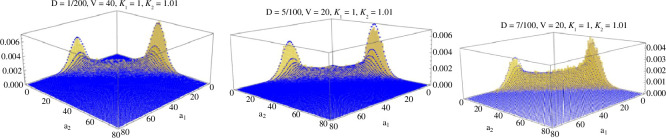
Histograms for the fraction of occupation times in all states a of X are shown in yellow with $\tilde{\Pi }(a)$ overlaid in blue. The parameters are $\lambda_{1}^{\prime }=\lambda_{2}^{\prime }=D,\kappa_{1}^{\prime }=1,\kappa_{2}^{\prime }=1.01$ and V=20 was fixed while D was varied D=2/100, 5/100, 7/100 from left to right.

On the other hand, DITs still exist but are less pronounced if we keep DV<d but we increase the rates of inflows $\lambda_{i}^{\prime }$ and rate of outflows $\delta^{\prime }$. In this case, reactions $\emptyset \overset{\lambda_{i}}{\to }A_{i}$ and $A_{i}\overset{\delta }{\to }\emptyset$ compete with the effects of autocatalytic rates in either direction. The modes become less pronounced as the process spends an equal amount of time in the interior of E as in states $\{(0,n),(n,0){\}}_{n\geq 0}$. Figure 7 shows the effect of decreasing the inflow and outflow rates while keeping the volume fixed and shows the empirical long-term occupation time of the simulated Markov chain X along with analytic plots of $\tilde{\Pi }$. We note that despite the significant effect of the inflow and outflow reactions, our distribution $\tilde{\Pi }$ matches the empirical estimates of Π well on all of E. This agrees with our numerical conclusions about $B^{*}(a)\approx 0$ for all a∈E.

## 4. Discussion

In this article, we studied a family of general autocatalytic reaction systems comprised of bimolecular interactions and plus inflows and outflows of molecular species from the system. The stochastic Markov chain model for the system is known to be ergodic with a unique stationary distribution, but the exact form of this distribution is only known in the case of symmetric autocatalytic rates. To investigate the asymmetric case, we proposed an approximate stationary distribution $\tilde{\Pi }$ in an explicit form that is a weighted version of the distribution from the symmetric case. This distribution is known to be the stationary distribution of a related model in population genetics which is a conserved system with no inflows and outflows.

We derived an error function $B^{*}$ which measures how close the proposed distribution is to the true stationary one. Numerical evaluations of this function, performed for a system with d=2 species, show that our proposed distribution matches the true stationary distribution for almost all values in the state space (and is very close to it in the exceptional values). This is also confirmed by comparison of the empirical distribution of long-term fraction of occupation times for the Markov chain with analytic values of the proposed distribution.

The explicit form we provide for the proposed distribution $\tilde{\Pi }$ allows us to show that, under the usual mass-action scaling of the system using the order of magnitude of the overall species count as a parameter, the phenomenon of DITs holds in the same parameter regime as in the symmetric case. The asymmetry of the autocatalytic rates produces a significant asymmetry in the modes of the stationary distribution, even under very small rate differences. The magnitude of inflow and outflow rates relative to the autocatalytic rates is shown to determine the extent to which DITs are felt in the system, with the speed of transitions decreasing as the system becomes more open.

A heuristic correspondence between outflows and inflows of the autocatalytic reaction system led us to the Moran model with genic selection. As this correspondence does not respect the evolution of values for the total number of molecules in the system, it does not appear useful in finite time. In the long time limit, with symmetry in the autocatalytic rates, we get equal relative occupation times in the original and the transformed system. Once the symmetry in these rates is broken, a small discrepancy is noticed but only at the values at the boundaries of the state space. It would be useful to further understand whether this discrepancy is a real feature or not. Unfortunately, the change of measure that maps the neutral Moran to one with genic selection does not apply directly to a model with fluctuating total number of molecules, so one would have to explore how one could interchange the limit of taking the infinite time horizon with conditioning on the total system size. Since the total mass process does not depend on autocatalytic rates, this could unify the results in the symmetric and asymmetric cases.

In terms of other work, we would like to note some examples of the type of reaction systems where our approximation of the stationary distribution could be useful. Several recent studies of chromatin modification circuits responsible for dictating epigenetic cell memory reflect on important experimental work. These mathematical studies [25–28] analyse stochastic models of an autocatalytic reaction system, in order to derive the temporal duration of the cell identity, and they note the importance of asymmetry in the system between repressed and active gene states. To analyse the model, the authors use a timescale separation of reactions in the system and develop a novel singular perturbation approach to derive the approximate stationary distribution of this autocatalytic system. Because of the inherent stochastic switching feature and asymmetry in the autocatalysis rates, one could try to see if our framework can be tailored to derive a different approximation for the stationary distribution and compare them to the existing approximation. This future work would provide a real application of our method to experimental studies.

## Data Availability

The paper uses simulated data generated by a stochastic simulation algorithm. We used Mathematica code to implement a standard Gillespie algorithm for the chemical reaction network modelled by a continuous Markov chain. We have provided the complete Mathematica code for the algorithm we used within electronic supplementary material file titled ‘code.pdf’ as well as in native format in electronic supplementary material file titled ‘GillespieSSa.nb’ [29]. The code is also published at Zenodo [30].

## Appendix A

We start from

$$\begin{array}{rl} & B^{*}(a)=\frac{1}{{\tilde{\pi }}_{n}(a)}\;[(L_{n-1}+L_{n}+L_{n+1})\;(a)-R_{n}\;(a)]\\ \text{with} & \\ & R_{n}\;(a)=[\lambda_{1}+\lambda_{2}+n\delta +(\kappa_{1}+\kappa_{2})\;a_{1}a_{2}]\;{\tilde{\pi }}_{n}\;(a),\\ & L_{n-1}(a)=\frac{n\delta \lambda_{1}}{\lambda_{1}+\lambda_{2}}{\tilde{\pi }}_{n-1}(a-e_{1})+\frac{n\delta \lambda_{2}}{\lambda_{1}+\lambda_{2}}{\tilde{\pi }}_{n-1}(a-e_{2}),\\ & L_{n}(a)=\kappa_{2}(a_{1}+1)(a_{2}-1){\tilde{\pi }}_{n}(a+e_{1}-e_{2})+\kappa_{1}(a_{1}-1)(a_{2}+1){\tilde{\pi }}_{n}(a-e_{1}+e_{2}),\\ & L_{n+1}(a)=\frac{\lambda_{1}+\lambda_{2}}{n+1}(a_{1}+1){\tilde{\pi }}_{n+1}(a+e_{1})+\frac{\lambda_{1}+\lambda_{2}}{n+1}(a_{2}+1){\tilde{\pi }}_{n+1}(a+e_{2}). \end{array}$$

We use our expression (3.14) (dropping the subscripts in the Gauss hypergeometric function ${,}_{2}F_{1}$):

π~n(i)=1F(−n,α1;1−α2−n;κ1κ2)(κ1κ2)i(ni)Γ(α1+i)Γ(α2+n−i)Γ(α1)Γ(α2+n),

to get

$$\begin{array}{rl} \frac{{\tilde{\pi }}_{n-1}(a-e_{1})}{{\tilde{\pi }}_{n}(a)} & =\frac{a_{1}}{n}\frac{n-1+\alpha_{2}}{a_{1}-1+\alpha_{1}}\frac{\kappa_{2}}{\kappa_{1}}\frac{F(-n,\alpha_{1},1-\alpha_{2}-n,\frac{\kappa_{1}}{\kappa_{2}})}{F(1-n,\alpha_{1},2-\alpha_{2}-n,\frac{\kappa_{1}}{\kappa_{2}})}\\ \frac{{\tilde{\pi }}_{n-1}(a-e_{2})}{{\tilde{\pi }}_{n}(a)} & =\frac{a_{2}}{n}\frac{n-1+\alpha_{2}}{a_{2}-1+\alpha_{2}}\frac{F(-n,\alpha_{1},1-\alpha_{2}-n,\frac{\kappa_{1}}{\kappa_{2}})}{F(1-n,\alpha_{1},2-\alpha_{2}-n,\frac{\kappa_{1}}{\kappa_{2}})}\\ \frac{{\tilde{\pi }}_{n}(a+e_{1}-e_{2})}{{\tilde{\pi }}_{n}(a)} & =\frac{a_{2}}{a_{1}+1}\frac{a_{1}+\alpha_{1}}{a_{2}-1+\alpha_{2}}\frac{\kappa_{1}}{\kappa_{2}}\\ \frac{{\tilde{\pi }}_{n}(a-e_{1}+e_{2})}{{\tilde{\pi }}_{n}(a+e_{1}-e_{2})} & =\frac{a_{1}}{a_{2}+1}\frac{a_{2}+\alpha_{2}}{a_{1}-1+\alpha_{1}}\frac{\kappa_{2}}{\kappa_{1}}\\ \frac{{\tilde{\pi }}_{n+1}(a+e_{1})}{{\tilde{\pi }}_{n}(a)} & =\frac{n+1}{a_{1}+1}\frac{a_{1}+\alpha_{1}}{n+\alpha_{2}}\frac{\kappa_{1}}{\kappa_{2}}\frac{F(-n,\alpha_{1},1-\alpha_{2}-n,\frac{\kappa_{1}}{\kappa_{2}})}{F(-1-n,\alpha_{1},-\alpha_{2}-n,\frac{\kappa_{1}}{\kappa_{2}})}\\ \frac{{\tilde{\pi }}_{n+1}(a)}{{\tilde{\pi }}_{n}(a)} & =\frac{n+1}{a_{2}+1}\frac{a_{2}+\alpha_{2}}{n+\alpha_{2}}\frac{F(-n,\alpha_{1},1-\alpha_{2}-n,\frac{\kappa_{1}}{\kappa_{2}})}{F(-1-n,\alpha_{1},-\alpha_{2}-n,\frac{\kappa_{1}}{\kappa_{2}})}. \end{array}$$

Then $L_{n-1},L_{n},L_{n+1}$ divided by ${\tilde{\pi }}_{n}$ become

$$\begin{array}{rl} & \frac{L_{n-1}}{{\tilde{\pi }}_{n}(a)}=\frac{(n-1+\alpha_{2})\delta }{\lambda_{1}+\lambda_{2}}\frac{F(-n,\alpha_{1},1-\alpha_{2}-n,\frac{\kappa_{1}}{\kappa_{2}})}{F(1-n,\alpha_{1},2-\alpha_{2}-n,\frac{\kappa_{1}}{\kappa_{2}})}[\frac{a_{1}\lambda_{1}}{a_{1}-1+\alpha_{1}}\frac{\kappa_{2}}{\kappa_{1}}+\frac{a_{2}\lambda_{2}}{a_{2}-1+\alpha_{2}}]\\ & \frac{L_{n}}{{\tilde{\pi }}_{n}(a)}=\kappa_{1}\frac{a_{2}-1}{a_{2}-1+\alpha_{2}}(a_{1}+\alpha_{1})a_{2}+\kappa_{2}\frac{a_{1}-1}{a_{1}-1+\alpha_{1}}(a_{2}+\alpha_{2})a_{1}\\ & \frac{L_{n+1}}{{\tilde{\pi }}_{n}(a)}=\frac{\lambda_{1}+\lambda_{2}}{\left(n+\alpha_{2}\right)}\frac{F(-n,\alpha_{1},1-\alpha_{2}-n,\frac{\kappa_{1}}{\kappa_{2}})}{F(-1-n,\alpha_{1},-\alpha_{2}-n,\frac{\kappa_{1}}{\kappa_{2}})}[\frac{\kappa_{1}}{\kappa_{2}}(a_{1}+\alpha_{1})+(a_{2}+\alpha_{2})]. \end{array}$$

We use the fact that $\alpha_{i}=\frac{\delta }{\lambda_{1}+\lambda_{2}}\frac{\lambda_{i}}{\kappa_{i}}$ (so $\sum_{i=1}^{2}\kappa_{i}\alpha_{i}=\delta$), $a_{1}+a_{2}=n$, and express $\frac{L_{n-1}}{{\tilde{\pi }}_{n}}$ as

(A 1)
$$\frac{L_{n-1}}{{\tilde{\pi }}_{n}(a)}=(n-1+\alpha_{2})\frac{F\left(-n,\alpha_{1},1-\alpha_{2}-n,\frac{\kappa_{1}}{\kappa_{2}}\right)}{F\left(1-n,\alpha_{1},2-\alpha_{2}-n,\frac{\kappa_{1}}{\kappa_{2}}\right)}\kappa_{2}[\frac{a_{1}\alpha_{1}}{a_{1}-1+\alpha_{1}}+\frac{a_{2}\alpha_{2}}{a_{2}-1+\alpha_{2}}].$$

For $\frac{L_{n}}{{\tilde{\pi }}_{n}(a)}$ we arrange terms as follows:

$$\begin{array}{rl} \frac{L_{n}}{{\tilde{\pi }}_{n}(a)}= & \sum_{i=1}^{2}\sum_{j\neq i}\kappa_{i}(a_{i}+\alpha_{i})\frac{a_{j}-1}{a_{j}-1+\alpha_{j}}a_{j}\\ = & \sum_{i=1}^{2}\sum_{j\neq i}\kappa_{i}(a_{i}+\alpha_{i})a_{j}-\sum_{i=1}^{2}\sum_{j\neq i}\frac{\kappa_{i}(a_{i}+\alpha_{i})a_{j}\alpha_{j}}{a_{j}-1+\alpha_{j}}\\ = & (\kappa_{1}+\kappa_{2})a_{1}a_{2}+\sum_{i=1}^{2}\sum_{j\neq i}\kappa_{i}\alpha_{i}a_{j}-\sum_{i=1}^{2}\sum_{j\neq i}\frac{\kappa_{i}(a_{i}+\alpha_{i})a_{j}\alpha_{j}}{a_{j}-1+\alpha_{j}}\\ = & (\kappa_{1}+\kappa_{2})a_{1}a_{2}+\sum_{i=1}^{2}\sum_{j=1}^{2}\kappa_{i}\alpha_{i}a_{j}-\sum_{i=1}^{2}\kappa_{i}\alpha_{i}a_{i}-\sum_{i=1}^{2}\sum_{j=1}^{2}\frac{\kappa_{j}(a_{j}+\alpha_{j})a_{i}\alpha_{i}}{a_{i}-1+\alpha_{i}}+\sum_{i=1}^{2}\frac{\kappa_{i}(a_{i}+\alpha_{i})a_{i}\alpha_{i}}{a_{i}-1+\alpha_{i}}\\ = & (\kappa_{1}+\kappa_{2})a_{1}a_{2}+n\delta -\sum_{i=1}^{2}\kappa_{i}\alpha_{i}a_{i}-\sum_{i=1}^{2}\sum_{j=1}^{2}\frac{\kappa_{j}(a_{j}+\alpha_{j})a_{i}\alpha_{i}}{a_{i}-1+\alpha_{i}}+\sum_{i=1}^{2}\kappa_{i}a_{i}\alpha_{i}+\sum_{i=1}^{2}\frac{\kappa_{i}a_{i}\alpha_{i}}{a_{i}-1+\alpha_{i}}\\ = & (\kappa_{1}+\kappa_{2})a_{1}a_{2}+n\delta -\sum_{i=1}^{2}\sum_{j=1}^{2}\frac{\kappa_{j}(a_{j}+\alpha_{j})a_{i}\alpha_{i}}{a_{i}-1+\alpha_{i}}+\sum_{i=1}^{2}\frac{\kappa_{i}a_{i}\alpha_{i}}{a_{i}-1+\alpha_{i}}\\ = & (\kappa_{1}+\kappa_{2})a_{1}a_{2}+n\delta -(\sum_{j=1}^{2}\kappa_{j}(a_{j}+\alpha_{j}))\sum_{i=1}^{2}\frac{a_{i}\alpha_{i}}{a_{i}-1+\alpha_{i}}+\sum_{i=1}^{2}\frac{\kappa_{i}a_{i}\alpha_{i}}{a_{i}-1+\alpha_{i}}\\ \; \end{array}$$

so that the first two terms will cancel the last two terms in $\frac{R_{n}}{{\tilde{\pi }}_{n}}$; for the remaining terms, we have

$$\begin{array}{rl} \; & \sum_{i=1}^{2}\frac{\kappa_{i}a_{i}\alpha_{i}}{a_{i}-1+\alpha_{i}}-(\sum_{j=1}^{2}\kappa_{j}(a_{j}+\alpha_{j}))\sum_{i=1}^{2}\frac{a_{i}\alpha_{i}}{a_{i}-1+\alpha_{i}}\\ & =\;\kappa_{1}\frac{a_{1}\alpha_{1}}{a_{1}-1+\alpha_{1}}+\kappa_{2}\frac{a_{2}\alpha_{2}}{a_{2}-1+\alpha_{2}}\\ & \;\;-\kappa_{1}(a_{1}+\alpha_{1})\frac{a_{1}\alpha_{1}}{a_{1}-1+\alpha_{1}}-\kappa_{2}(a_{2}+\alpha_{2})\frac{a_{1}\alpha_{1}}{a_{1}-1+\alpha_{1}}\\ & \;\;-\kappa_{1}(a_{1}+\alpha_{1})\frac{a_{2}\alpha_{2}}{a_{2}-2+\alpha_{2}}-\kappa_{2}(a_{2}+\alpha_{2})\frac{a_{2}\alpha_{2}}{a_{2}-1+\alpha_{2}}\\ & =\;\kappa_{2}\frac{a_{1}\alpha_{1}}{a_{1}-1+\alpha_{1}}[\frac{\alpha_{2}}{\alpha_{1}}-\frac{\alpha_{2}}{\alpha_{1}}(a_{1}+\alpha_{1})-(a_{2}+\alpha_{2})]+\kappa_{2}\frac{a_{2}\alpha_{2}}{a_{2}-1+\alpha_{2}}[1-\frac{\alpha_{2}}{\alpha_{1}}(a_{1}+\alpha_{1})-(a_{2}+\alpha_{2})]\\ & =\;\kappa_{2}\frac{a_{1}\alpha_{1}}{a_{1}-1+\alpha_{1}}[\frac{\alpha_{2}}{\alpha_{1}}-\frac{\alpha_{2}}{\alpha_{1}}a_{1}-a_{2}-2\alpha_{2}]+\kappa_{2}\frac{a_{2}\alpha_{2}}{a_{2}-1+\alpha_{2}}[1-\frac{\alpha_{2}}{\alpha_{1}}a_{1}-a_{2}-2\alpha_{2}]\\ & =\;\kappa_{2}\frac{a_{1}\alpha_{1}}{a_{1}-1+\alpha_{2}}[\frac{\alpha_{2}}{\alpha_{1}}+a_{1}-\frac{\alpha_{2}}{\alpha_{1}}a_{1}-1-\alpha_{2}]+\kappa_{2}\frac{a_{2}\alpha_{2}}{a_{2}-1+\alpha_{2}}[a_{1}-\frac{\alpha_{2}}{\alpha_{1}}a_{1}-\alpha_{2}]\\ & \;\;-\kappa_{2}(n-1+\alpha_{2})\sum_{i=1}^{2}\frac{a_{i}\alpha_{i}}{a_{i}-1+\alpha_{i}}\\ & =\;\kappa_{2}\sum_{i=1}^{2}\frac{a_{i}\alpha_{i}}{a_{i}-1+\alpha_{i}}[a_{1}(1-\frac{\alpha_{2}}{\alpha_{1}})-\alpha_{2}]+\kappa_{2}\frac{a_{1}\alpha_{1}}{a_{1}-1+\alpha_{1}}[\frac{\alpha_{2}}{\alpha_{1}}-1]-\kappa_{2}(n-1+\alpha_{2})\sum_{i=1}^{2}\frac{a_{i}\alpha_{i}}{a_{i}-1+\alpha_{i}}\\ & =\;\sum_{i=1}^{2}\frac{a_{i}\alpha_{i}}{a_{i}-1+\alpha_{i}}[a_{1}(\kappa_{2}-\kappa_{1})-\kappa_{2}\alpha_{2}]+\frac{a_{1}\alpha_{1}}{a_{1}-1+\alpha_{1}}[\kappa_{1}-\kappa_{2}]-\kappa_{2}(n-1+\alpha_{2})\sum_{i=1}^{2}\frac{a_{i}\alpha_{i}}{a_{i}-1+\alpha_{i}} \end{array}$$

so that

(A 2)
$$\begin{array}{l} \begin{array}{rl} \frac{L_{n}}{{\tilde{\pi }}_{n}(a)}= & (\kappa_{1}+\kappa_{2})a_{1}a_{2}+n\delta -\kappa_{2}(n-1+\alpha_{2})\sum_{i=1}^{2}\frac{a_{i}\alpha_{i}}{a_{i}-1+\alpha_{i}}\\ & +\sum_{i=1}^{2}\frac{a_{i}\alpha_{i}}{a_{i}-1+\alpha_{i}}[a_{1}(\kappa_{2}-\kappa_{1})-\alpha_{2}]+\frac{a_{1}\alpha_{1}}{a_{1}-1+\alpha_{1}}[\kappa_{1}-\kappa_{2}], \end{array} \end{array}$$

where the first two terms will cancel with terms in $\frac{R_{n}}{{\tilde{\pi }}_{n}}$, and the third term is the same as in $\frac{L_{n-1}}{{\tilde{\pi }}_{n}}$ from (A 1) apart from the ratio of hypergeometric functions.

Finally, we express $\frac{L_{n+1}}{{\tilde{\pi }}_{n}}$ as

$$\begin{array}{r} \begin{array}{rl} \frac{L_{n+1}}{{\tilde{\pi }}_{n}(a)} & =\frac{\lambda_{1}+\lambda_{2}}{n+\alpha_{2}}\frac{F(-n,\alpha_{1},1-\alpha_{2}-n,\frac{\kappa_{1}}{\kappa_{2}})}{F(-1-n,\alpha_{1},-\alpha_{2}-n,\frac{\kappa_{1}}{\kappa_{2}})}[\frac{\kappa_{1}}{\kappa_{2}}(a_{1}+\alpha_{1})+(a_{2}+\alpha_{2})]\\ & =\frac{\lambda_{1}+\lambda_{2}}{n+\alpha_{2}}\frac{F(-n,\alpha_{1},1-\alpha_{2}-n,\frac{\kappa_{1}}{\kappa_{2}})}{F(-1-n,\alpha_{1},-\alpha_{2}-n,\frac{\kappa_{1}}{\kappa_{2}})}[(n+\alpha_{2})+\frac{\kappa_{1}}{\kappa_{2}}(a_{1}+\alpha_{1})-a_{1}]\\ & =(\lambda_{1}+\lambda_{2})\frac{F(-n,\alpha_{1},1-\alpha_{2}-n,\frac{\kappa_{1}}{\kappa_{2}})}{F(-1-n,\alpha_{1},-\alpha_{2}-n,\frac{\kappa_{1}}{\kappa_{2}})}+\frac{\lambda_{1}+\lambda_{2}}{n+\alpha_{2}}\frac{F(-n,\alpha_{1},1-\alpha_{2}-n,\frac{\kappa_{1}}{\kappa_{2}})}{F(-1-n,\alpha_{1},-\alpha_{2}-n,\frac{\kappa_{1}}{\kappa_{2}})}[a_{1}(\frac{\kappa_{1}}{\kappa_{2}}-1)+\alpha_{2}]. \end{array} \end{array}$$

For $B^{*}=\frac{1}{{\tilde{\pi }}_{n}}(L_{n-1}+L_{n}-L_{n-1}-R_{n})$, we now get the expression in (3.15):

$$\begin{array}{r} \begin{array}{rl} B^{*}(a)= & (\lambda_{1}+\lambda_{2})[1-\frac{F(-n,\alpha_{1},1-\alpha_{2}-n,\frac{\kappa_{1}}{\kappa_{2}})}{F(-1-n,\alpha_{1},-\alpha_{2}-n,\frac{\kappa_{1}}{\kappa_{2}})}]\\ & -\kappa_{2}(n-1+\alpha_{2})\sum_{i=1}^{2}\frac{a_{i}\alpha_{i}}{a_{i}-1+\alpha_{i}}[\frac{F(-n,\alpha_{1},1-\alpha_{2}-n,\frac{\kappa_{1}}{\kappa_{2}})}{F(1-n,\alpha_{1},2-\alpha_{2}-n,\frac{\kappa_{1}}{\kappa_{2}})}-1]\\ & -\sum_{i=1}^{2}\frac{a_{i}\alpha_{i}}{a_{i}-1+\alpha_{i}}[a_{1}(\kappa_{2}-\kappa_{1})-\kappa_{2}\alpha_{2}]-\frac{a_{1}\alpha_{1}}{a_{1}-1+\alpha_{1}}[\kappa_{1}-\kappa_{2}]\\ & -\frac{\lambda_{1}+\lambda_{2}}{n+\alpha_{2}}\frac{F(-n,\alpha_{1},1-\alpha_{2}-n,\frac{\kappa_{1}}{\kappa_{2}})}{F(-1-n,\alpha_{1},-\alpha_{2}-n,\frac{\kappa_{1}}{\kappa_{2}})}[a_{1}(\frac{\kappa_{1}}{\kappa_{2}}-1)+\alpha_{2}]. \end{array} \end{array}$$

## Appendix B

We start from (3.15) and use the (3.16) scaling by V

$$\kappa_{i}=\frac{\kappa_{i}^{\prime }}{V},\;\;\lambda_{i}=DV,\;\;\delta =D,\;\;\;\Rightarrow \;\alpha_{i}=\frac{DV}{d\kappa_{i}^{\prime }}=V\alpha_{i}^{\prime }$$

to get

$$\begin{array}{r} \begin{array}{rl} B_{V}^{*}(a)= & dDV[1-\frac{F(-n,V\alpha_{1}^{\prime },1-V\alpha_{2}^{\prime }-n,\frac{\frac{\kappa_{1}^{\prime }}{V}}{\frac{\kappa_{2}^{\prime }}{V}})}{F(-1-n,V\alpha_{1}^{\prime },-V\alpha_{2}^{\prime }-n,\frac{\frac{\kappa_{1}^{\prime }}{V}}{\frac{\kappa_{2}^{\prime }}{V}})}]\\ & -\frac{\kappa_{2}^{\prime }}{V}(n-1+V\alpha_{2}^{\prime })\sum_{i=1}^{2}\frac{a_{i}V\alpha_{i}^{\prime }}{a_{i}-1+V\alpha_{i}^{\prime }}[\frac{F(-n,V\alpha_{1}^{\prime },1-V\alpha_{2}^{\prime }-n,\frac{\frac{\kappa_{1}^{\prime }}{V}}{\frac{\kappa_{2}^{\prime }}{V}})}{F(1-n,V\alpha_{1}^{\prime },2-V\alpha_{2}^{\prime }-n,\frac{\frac{\kappa_{1}^{\prime }}{V}}{\frac{\kappa_{2}^{\prime }}{V}})}-1]\\ & -\sum_{i=1}^{2}\frac{a_{i}V\alpha_{i}^{\prime }}{a_{i}-1+VV\alpha_{i}^{\prime \prime }}[a_{1}(\frac{\kappa_{2}^{\prime }}{V}-\frac{\kappa_{1}^{\prime }}{V})-\frac{\kappa_{2}^{\prime }}{V}V\alpha_{2}^{\prime }]-\frac{a_{1}V\alpha_{1}^{\prime }}{a_{1}-1+V\alpha_{1}^{\prime }}[\frac{\kappa_{1}^{\prime }}{V}-\frac{\kappa_{2}^{\prime }}{V}]\\ & -\frac{\lambda_{1}+\lambda_{2}}{n+V\alpha_{2}^{\prime }}\frac{F(-n,V\alpha_{1}^{\prime },1-V\alpha_{2}^{\prime }-n,\frac{\frac{\kappa_{1}^{\prime }}{V}}{\frac{\kappa_{2}^{\prime }}{V}})}{F(-1-n,V\alpha_{1}^{\prime },-V\alpha_{2}^{\prime }-n,\frac{\frac{\kappa_{1}^{\prime }}{V}}{\frac{\kappa_{2}^{\prime }}{V}})}[a_{1}(\frac{\frac{\kappa_{1}^{\prime }}{V}}{\frac{\kappa_{2}^{\prime }}{V}}-1)+V\alpha_{2}^{\prime }] \end{array} \end{array}$$

$$\begin{array}{r} \begin{array}{rl} = & dDV[1-\frac{F(-n,V\alpha_{1}^{\prime },1-V\alpha_{2}^{\prime }-n,\frac{\kappa_{1}^{\prime }}{\kappa_{2}^{\prime }})}{F(-1-n,V\alpha_{1}^{\prime },-V\alpha_{2}^{\prime }-n,\frac{\kappa_{1}^{\prime }}{\kappa_{2}^{\prime }})}]\\ & -\kappa_{2}(n-1+V\alpha_{2}^{\prime })\sum_{i=1}^{2}\frac{a_{i}\alpha_{i}^{\prime }}{a_{i}-1+V\alpha_{i}^{\prime }}[\frac{F(-n,V\alpha_{1}^{\prime },1-V\alpha_{2}^{\prime }-n,\frac{\kappa_{1}^{\prime }}{\kappa_{2}^{\prime }})}{F(1-n,V\alpha_{1}^{\prime },2-V\alpha_{2}^{\prime }-n,\frac{\kappa_{1}^{\prime }}{\kappa_{2}^{\prime }})}-1]\\ & -\sum_{i=1}^{2}\frac{a_{i}\alpha_{i}^{\prime }}{a_{i}-1+V\alpha_{i}^{\prime }}[a_{1}(\kappa_{2}-\kappa_{1})-\kappa_{2}\alpha_{2}^{\prime }]-\frac{a_{1}\alpha_{1}^{\prime }}{a_{1}-1+V\alpha_{1}^{\prime }}[\kappa_{1}^{\prime }-\kappa_{2}^{\prime }]\\ & -\frac{dDV}{n+V\alpha_{2}^{\prime }}\frac{F(-n,V\alpha_{1}^{\prime },1-V\alpha_{2}^{\prime }-n,\frac{\kappa_{1}^{\prime }}{\kappa_{2}^{\prime }})}{F(-1-n,V\alpha_{1}^{\prime },-V\alpha_{2}^{\prime }-n,\frac{\kappa_{1}^{\prime }}{\kappa_{2}^{\prime }})}[a_{1}(\frac{\kappa_{1}^{\prime }}{\kappa_{2}^{\prime }}-1)+V\alpha_{2}^{\prime }]. \end{array} \end{array}$$

Substituting $\alpha_{i}^{\prime }=\frac{D}{d\kappa_{i}^{\prime }}$ and rearranging terms we get the expression in equation (3.17):

$$\begin{array}{r} \begin{array}{rl} B_{V}^{*}(a)= & dDV([1-\frac{F(-n,\frac{DV}{d\kappa_{1}^{\prime }},1-\frac{DV}{d\kappa_{2}^{\prime }}-n,\frac{\kappa_{1}^{\prime }}{\kappa_{2}^{\prime }})}{F(-1-n,\frac{DV}{d\kappa_{1}^{\prime }},-\frac{DV}{d\kappa_{2}^{\prime }}-n,\frac{\kappa_{1}^{\prime }}{\kappa_{2}^{\prime }})}]\\ + & \frac{1}{n+\frac{DV}{d\kappa_{2}^{\prime }}}\frac{F(-n,\frac{DV}{d\kappa_{1}^{\prime }},1-\frac{DV}{d\kappa_{2}^{\prime }}-n,\frac{\kappa_{1}^{\prime }}{\kappa_{2}^{\prime }})}{F(-1-n,\frac{DV}{d\kappa_{1}^{\prime }},-\frac{DV}{d\kappa_{2}^{\prime }}-n,\frac{\kappa_{1}^{\prime }}{\kappa_{2}^{\prime }})}[a_{1}(1-\frac{\kappa_{1}^{\prime }}{\kappa_{2}^{\prime }})-\frac{DV}{d\kappa_{2}^{\prime }}])\\ - & \kappa_{2}^{\prime }(n-1+\frac{DV}{d\kappa_{2}^{\prime }})[\frac{F(-n,\frac{DV}{d\kappa_{1}^{\prime }},1-\frac{DV}{d\kappa_{2}^{\prime }}-n,\frac{\kappa_{1}^{\prime }}{\kappa_{2}^{\prime }})}{F(1-n,\frac{DV}{d\kappa_{1}^{\prime }},2-\frac{DV}{d\kappa_{2}^{\prime }}-n,\frac{\kappa_{1}^{\prime }}{\kappa_{2}^{\prime }})}-1](\frac{a_{1}\frac{DV}{d\kappa_{1}^{\prime }}}{a_{1}-1+\frac{DV}{d\kappa_{1}^{\prime }}}+\frac{a_{2}\frac{DV}{d\kappa_{2}^{\prime }}}{a_{2}-1+\frac{DV}{d\kappa_{2}^{\prime }}})\\ - & \kappa_{2}^{\prime }[a_{1}(1-\frac{\kappa_{1}^{\prime }}{\kappa_{2}^{\prime }})-\frac{DV}{d}](\frac{a_{1}\frac{DV}{d\kappa_{1}^{\prime }}}{a_{1}-1+\frac{DV}{d\kappa_{1}^{\prime }}}+\frac{a_{2}\frac{DV}{d\kappa_{2}^{\prime }}}{a_{2}-1+\frac{DV}{d\kappa_{2}^{\prime }}})+\kappa_{2}^{\prime }\frac{a_{1}\frac{D}{d\kappa_{1}^{\prime }}}{a_{1}-1+\frac{DV}{d\kappa_{1}^{\prime }}}[1-\frac{\kappa_{1}^{\prime }}{\kappa_{2}^{\prime }}]. \end{array} \end{array}$$
